# Prevalence of Antimicrobial Resistance and Virulence Gene Elements of *Salmonella* Serovars From Ready-to-Eat (RTE) Shrimps

**DOI:** 10.3389/fmicb.2019.01613

**Published:** 2019-07-11

**Authors:** Abeni Beshiru, Isoken H. Igbinosa, Etinosa O. Igbinosa

**Affiliations:** ^1^Applied Microbial Processes and Environmental Health Research Group, Department of Microbiology, Faculty of Life Sciences, University of Benin, Benin City, Nigeria; ^2^Department of Environmental Management and Toxicology, Faculty of Life Sciences, University of Benin, Benin City, Nigeria; ^3^Sustainable Development Office, University of Benin, Benin City, Nigeria

**Keywords:** multidrug resistant, salmonellosis, virulence determinants, seafood, health risk

## Abstract

Gastrointestinal illnesses continue to be a global public health risk. Exposure to foodborne *Salmonella* directly or indirectly through consumption of ready-to-eat seafood can be an important route of infection to humans. This study was designed to estimate the population cell density, prevalence, virulence gene signatures, and antibiotic resistance of *Salmonella* serovars from ready-to-eat shrimps. Ready-to-eat (RTE) shrimp samples were obtained from different open markets in Delta and Edo States, Nigeria from November 2016 to October 2017. We employed classical and polymerase chain reaction (PCR) approaches. The mean *Salmonella* species enumerated from the RTE shrimps ranged from −0.301 to 5.434 log_10_ cfu/g with 210/1440 (14.58%) of the RTE shrimp samples harbored *Salmonella* species. After biochemical and PCR approach, the identified isolates were *Salmonella* Enteritidis 11(24.4%), *Salmonella* Typhimurium 14 (31.1%) and other *Salmonella* spp. 20 (44.4%). All *Salmonella* species recovered were resistant to penicillin and erythromycin with 100% sensitivity to cefotaxime, cephalothin, colistin, and polymyxin B. Findings on the multidrug-resistant (MDR) profile showed that a total of 9/14 (64.3%) of *Salmonella* Enteritidis were resistant to 5 antibiotics which belongs to 3 different groups of antimicrobials with a multiple antibiotic-resistant (MAR) index of 0.21; while 3/11 (27.3%) of *Salmonella* Typhimurium were resistant to 11 antibiotics which belongs to 7 different groups of antimicrobials with a MAR index of 0.46. Virulence genes (*spi*A, *sip*B, *inv*A, *sif* A, *flj*B, and *sef*A) and resistance genes (class 1 and II integrase, *sul*2, *catB*3, *flor, tmp, bla*_TEM_, *str*B, *dfr*1, and *tet*C) were also detected in some of the *Salmonella* species with variable percentage. This study indicates that ready-to-eat shrimps are probable reservoirs harboring *Salmonella* strains. The identified *Salmonella* isolates which exhibited virulence determinants and antibiotic-resistant coupled with high MAR index constitute a consumer health risk to the communities.

## Introduction

Shrimps constitute a large proportion of crustaceans which varies in sizes (Orji et al., 2016) and have been described as the most significant seafood traded on a global scale (Oosterveer, 2006). The world shrimp production for both farmed and captured shrimp is ~6 million tons with 60% entering the world market. Shrimp has been reported to be the most essentially traded fishery product internationally as it translates to value. Yearly shrimp exports presently value above US$10 billion, or 16% of total fish product exports (Food Agriculture Organization of the United Nations, 2008). Shrimp makes up 20% value of exported fishery products for more than 20 years (CAC, 2002). Imports of shrimps into developed nations are responsible for about 40% trade of intra-developed countries, while approximately 60% comes from developing nations. From developing nation exports, 80% goes to developed nation with only 20% left behind (Josupeit, 2005). Shrimps are one of the important exported aquaculture products from the tropics. The interaction of microbial diversity that comes in connection with shrimps during harvesting and processing is a prospective public health threats as a consequence of disease and spoilage transmission.

The main disease causing serovars of *Salmonella enterica* subspecies *enterica* which are pathogenic to humans as a result of diverse seafood and non-seafood products include *Salmonella* Typhimurium and *Salmonella* Enteritidis (Ed-dra et al., 2017). Salmonellosis which is an infection of the intestinal epithelium is instigated by the *Salmonella* genus (Igbinosa and Beshiru, 2017; Beshiru et al., 2018). Within the United States more than 40,000 cases of salmonellosis are recounted yearly with seafood considered as one of the most significant source of *Salmonella* (Brands et al., 2005; Duran and Marshall, 2005). Contamination results when the salmonellae enter RTE food and replicate within the food, as a result of inadequate food preparation, poor storage temperatures, or cross-contamination (Skyberg et al., 2006). Hence, the occurrence of *Salmonella* in RTE seafood from open market is an important food safety risk.

Antimicrobial-resistant *Salmonella* serovars may result from the continuous usage of antimicrobials in food animal production, where these antimicrobial resistant *Salmonella* are therefore disseminated to humans, usually through contaminated food. The application of antimicrobials in aquaculture systems has led to the accumulation of antibiotic-resistance genes and antibiotic-resistant bacteria (Yano et al., 2011; Igbinosa, 2016). Antibiotics commonly used in agricultural/aquaculture systems in Nigeria are gentamycin, ivermectin, oxytetracycline, tylosin, septinomycin, and cephalosporin. Food and Drug Administration (FDA) has permitted the use of five different drugs (sulfamerazine, chorionic gonadotropin, florfenicol, oxytetracycline hydrochloride, oxytetracycline dihydrate, as well as combination of sulfadimethoxine and ormetoprim) in aquaculture so long as the seafood harbors less than the required maximum residue limit (Serrano, 2005). FDA has also approved two drugs: hydrogen peroxide and formalinwith no tolerance level set a (Serrano, 2005). Multidrug resistance (MDR) in *Salmonella* is of significant concern as treatment regimens may be challenging, thus making management of these disease difficult. *Salmonella* Typhimurium is one of the most widespread MDR *Salmonella* serovars recovered from humans and animals in the United States (Brunelle et al., 2013). The continuous rise and dissemination of antibiotic resistance phenotypes and determinants among *Salmonella* serovars has metamorphosed into a public health space. Notably, strains of *Salmonella* which have clinically phenotypic and genotypic resistance to antibiotic agents such as extended spectrum cephalosporins and fluoroquinolones, have been recovered from food animals (Li et al., 2013; Igbinosa, 2015). Within developing countries, overuse and misuse of antibiotics has led to the upsurge of MDR in *Salmonella* strains (Ed-dra et al., 2017).

Antibiotic-resistant *Salmonella* connected with cultivated *Litopenaeus vannamei* have been reported in Malaysia where *Salmonella enterica* serovar Corvallis recovered from shrimp revealed multiple and individual antibiotic resistance profiles (Banerjee et al., 2012). In Nigeria there are some reports that have revealed the occurrence of *Salmonella* species from numerous food types, with no study on the surveillance of *Salmonella* Typhimurium, *Salmonella* Enteritidis and other *Salmonella* spp. from RTE shrimps. Hence, the objective of this research was to determine the prevalence, multiple antibiotic resistance, virulence and antibiotic resistance genes of *Salmonella* serovars recovered from retail RTE-shrimps in Nigeria.

## Materials and Methods

### Study Area

The RTE shrimp were obtained from major open markets in Delta and Edo States, Nigeria. There are 3 Senatorial Districts in each of Edo and Delta State. Six different markets were assessed from each state which makes it a total of 12 markets with 2 markets from each Senatorial District. In Delta State, markets include Ughelli main market, Sapele market (Delta Central Senatorial District), Ogbegonogo market, Ashafor market Aniocha Asaba market (Delta North Senatorial District), main market Oleh Isoko, and Igbudu market Warri (Delta South Senatorial District). For Edo State, markets include New Benin market, Oba market (Edo South Senatorial District), Igarra market, Jattu market (Edo North Senatorial District), Uromi main market and Ekpoma market (Edo Central Senatorial District). The respective markets were chosen based on the strategic locations in their respective communities and are highly dense due to the population of individuals that patronizes these markets. The RTE shrimp that were collected from these markets were mainly tiger shrimps (*Penaeus monodon*) and pink shrimp (*Penaeus notialis*) and included smoked shrimps, dried shrimps, fried shrimps, sauced shrimps, and boiled shrimps.

### Sample Collection

One thousand four hundred and forty RTE shrimp samples were obtained between November 2016 and October, 2017. Ten samples each were obtained from each of the respective 12 selected open markets (6 each from Delta and Edo States) culminating in the 1440 RTE samples. Samples were obtained based on the type of RTE shrimps available with respect to the sampling location. The RTE shrimp samples were obtained from the selected open markets with the aid of a sterile polythene bag. The polythene bags were immediately placed on ice pack and conveyed to the laboratory where microbiological analyses were carried out within 24 h after collection.

### Enrichment, Enumeration and Isolation of *Salmonella* Species

This was carried out in line with the International Organization for Standardization (2017). Twenty-five grams of individual RTE shrimp samples was weighed and placed in a sterile homogenizer bag containing 225 mL of tryptone soy broth (TSB) (Merck, Darmstadt, Germany), as pre-enrichment and incubated at 37°C for 18–24 h. Before incubation, the stock suspension was serially diluted using sterile distilled water from 10^−1^ to 10^−9^. Dilution with 100 μL of each diluent aseptically platted in triplicates into xylose lysine deoxycholate (XLD) agar (Lab M, Lancashire, United Kingdom) and hektoen enteric agar (HEA) (Lab M, Lancashire, United Kingdom). This was followed with incubated at 37°C for 24–48 h where presumptive *Salmonella* species which appear as distinct green colonies with or without black centers were enumerated and expressed in log_10_ colony forming units per gram (log_10_ cfu/g). After incubation with the pre-enrichment broth with TSB, 100 μL were inoculated into 9.0 mL of selenite cysteine F Broth (Lab M, Lancashire, United Kingdom) and incubated at 37°C for 18–24 h. After incubation, 100 μL of the turbid suspension was inoculated into XLD and HEA and incubated at 37°C for 18–24 h where a maximum of 2 presumptive *Salmonella* colonies were selected and sub-cultured on a fresh XLD and HEA and incubated at 37°C for 18–24 h. Distinct colonies were further purified on tryptone soy agar (TSA) (Merck, Darmstadt, Germany) incubated at 37°C for 18–24 h. Isolates were transferred into a 1 mL TSA in an Eppendorf tube and incubated at 37°C for 24 h and stored in the refrigerator at 4°C until ready for further use.

### Presumptive Identification of *Salmonella* Species

All *Salmonella* species were screened via biochemical (oxidase, catalase, indole, and sugar fermentation test, citrate), morphological (Gram reaction with 3% KOH test), and cultural (colony) characterization. Analytical Profile Index 20E (API 20E) was also used for the *Salmonella* species respectively according to the manufacturer's instructions (BioMerieux, Marcy-l'Étoile, France) using API lab plus software (bioMerieux, Marcy l'Etoile, France).

### Genomic Deoxyribonucleic Acid (gDNA) Extraction Procedure

Genomic DNA from *Salmonella* species were extracted via boiling method described by Igbinosa et al. (2017). The *Salmonella* isolates were inoculated in 5.0 mL TSB incubated at 37°C for 18–24 h (Beshiru et al., 2018). A 100 μL of the turbid suspension was combined with 100 μL of sterilized distilled water in a 2.0 mL Eppendorf tube and subjected to a dry bath (MK200-2, Shanghai, China) for 15 min at 100°C for cell lyses. The lysed cell mixture was then centrifuged with a mini centrifuge (Mini 14k, Zhuhai, Guangdong, China) at 14 500 r/min for 15 min. The cell fragments were carefully separated from the supernatant. The supernatant was stored at −20°C as the template gDNA.

### Polymerase Chain Reaction Amplification Procedure

All reactions were carried out in 25.0 μL volume of reaction (10 × Buffer 2.5 μL; MgCl_2_ 1.0 μL; dNTP-Mix 3.0 μL; Taq polymerase 0.2 μL; Reverse primer 1.25 μL; Forward primer 1.25 μL; sterile double distilled H_2_0 10.8 μL and gDNA 5.0 μL). Primers used for the detection of *Salmonella* species are shown in Table S1. The reaction was performed via a Peltier-based Thermal Cycler (BioSeparation System, Shanxi, China) with an initial denaturation at 95°C for 10 min; 35 cycles of denaturation at 94°C for 60 s, primer annealing as indicated in Table S1 and extension at 72°C for 90 s; final extension at 72°C for 10 min. *Salmonella enterica* serovar Typhymurium ATCC 14028, *Salmonella* Enteritidis ATCC 13076, were used as positive controls while deionized water was used as a negative control for each test procedure. Thermal cyclic conditions for the detection of antibiotic-resistance genes for *Salmonella* species were as follows; initial denaturation at 94°C for 2 min followed by 35 cycles of 94°C for 1 min, annealing condition as in Table S2 and extension at 72°C for 1 min with a final extension at 72°C for 10 min and cooling to 4°C. The PCR conditions for amplification of the virulence genes were as follows: 5 min of initial denaturation at 95°C, followed by 30 cycles of denaturation at 94°C for 30 s, annealing as described in Table S3, and extension at 72°C for 60 s, ending with a final extension period of 72°C for 2 min. Electrophoresis of the amplified PCR products were loaded on 1.2% agarose gel (CLS-AG100, Warwickshire, United Kingdom) in 0.5 × TAE buffer (pH 8.5, 20 mM Na acetate, 40 mM Tris-HCl, 1 mM EDTA) and allowed to run for 1 h at 100 V. The gels were viewed via a UV transilluminator (EBOX VX5, Vilber Lourmat, France).

### Antimicrobial Susceptibility Profile of the *Salmonella* Isolates

Antimicrobial susceptibility profile of the *Salmonella* species was carried out using Kirby-Bauer disc diffusion method. Briefly, the purified isolates were inoculated in 5.0 mL Mueller-Hinton Broth (MHB) (Lab M, Lancashire, United Kingdom) and incubated overnight. The optical density (OD) of the turbidity of the broth was adjusted to McFarland standard 0.5 equivalent to 10^8^ cfu/mL. Using a sterile swab sticks, respective broth cultures were aseptically swabbed on Mueller Hinton Agar (Lab M, Lancashire, United Kingdom). A total of 24 antibiotic discs (Mast Diagnostics, Merseyside, United Kingdom) which included kanamycin (30 μg), gentamycin (10 μg), streptomycin (25 μg), erythromycin (15 μg), tobramycin (10 μg), ampicillin (10 μg), amoxicillin (25 μg), imipenem (10 μg), ampicillin/sulbactam (30 μg), meropenem (10 μg), cefotaxime (30 μg), sulfamethoxazole (30 μg), cephalothin (30 μg), trimethoprim (25 μg), erythromycin (15 μg), amoxicillin/clavulanate (30 μg), colistin (20 μg), chloramphenicol (30 μg), penicillin G (10 μnits), polymyxin B (300 units), oxytetracycline (30 μg), doxycycline (30 μg), tetracycline (30 μg), ofloxacin (5 μg), and ciprofloxacin (10 μg) were used for the susceptibility testing. The respective discs were also aseptically impregnated on the agar plates using a sterile forceps equidistant apart. Plates were allowed to stand at room temperature for 5 min and incubated at 37°C for 18–24 h. Resistance, intermediate or susceptibility profile of the isolates were elucidated by determining zone of inhibition and matched with the interpretative chart of Clinical Laboratory Standards Institute (2017) to determine the sensitivity, intermediate and resistance profiles of the isolates to the antibiotics used.

### Statistical Analysis

All data were analyzed using the statistical package SPSS (Version 21.0) and Microsoft Excel 2013. Descriptive statistics were carried out to determine the mean population density and expressed in Log_10_ CFU/g. One Way Analysis of Variance was applied to the densities from open markets while Duncan Multiple Range test was used to show significant difference between mean variables. The *p* < 0.05 were considered statistically significant.

## Results

### Population Cell Density of *Salmonella* Species From the RTE Shrimps

The mean *Salmonella* species counts from RTE shrimps obtained from open markets are presented in Table S4. The mean *Salmonella* species counts from the RTE shrimps are all expressed in log_10_ cfu/g. The values ranged from 0.079 to 3.516 (November), 0.613–3.817 (December), 0.255–4.492 (January), 0.602–4.841 (February), 0.959–4.822 (March), 1.562–5.118 (April), 1.573–5.434 (May), 2.003–5.274 (June), 2.001–5.356 (July), 1.782–4.555 (August), 0.944–4.754 (September), and −0.301 −3.748 (October) during the 12 month sampling regimen. Significant differences were observed across the respective markets as *p* < 0.01. For the respective markets, values ranged from 0.977 to 2.391 (Oba Market), 0.944–3.283 (New Benin Market), 0.613–3.231 (Jattu Market), 0.079–2.075 (Igarra Market), −0.301 −3.318 (Ekpoma Market), 1.272–3.484 (Uromi Market), 2.572–4.428 (Sapele Market), 3.053–4.481 (Ughele Market), 3.083–5.434 (Ogbegonogo Market), 3.185–5.205 (Ashafor Market), 3.161–4.435 (Igbudu Market), and 3.236–5.356 (Main Market, Oleh). Significant differences were observed across the respective months as *p* < 0.01.

### Prevalence of Positive *Salmonella* Samples

The distribution of positive *Salmonella* samples from respective markets include, 14/120 (11.7%) for Oba market, 10/120 (8.33%) for New Benin market, 8/120 (8.33%) for Jattu market, 9/120 (7.5%) for Igarra market, 7/120 (5.83%) for Ekpoma market, 10/120 (8.33%) for Uromi market, 23/120 (19.17%) for Sapele market, 27/120 (22.5%) for Ughele market, 26/120 (21.67%) for Ogbegonogo market, 25/120 (20.83%) for Ashafor market, 27/120 (22.5%) for Igbudu market, 24/120 (20%) for main Market Oleh. Overall, 210/1440 (14.58%) were positive for *Salmonella* species.

### *Salmonella* Detection From RTE Shrimps

This study revealed, 210/1440 (14.58 %) of the RTE shrimp samples were positive for *Salmonella* species. All the tentatively 210 *Salmonella* isolates were characterized via culture-based and biochemical procedures using Gram-reaction with 3% KOH test, oxidase, urease reactions, indole and motility tests. The *Salmonella* isolates that appear negative for urease, oxidase, indole and Gram-negative rods were selected as presumptive *Salmonella*. Only 67 *Salmonella* isolates were positive using this culture-based approach. Analytical profile index (API 20E) were further employed to confirmed the identity of 49 *Salmonella* isolates. From the 49 *Salmonella* isolates positive from the API test, *Salmonella* genus-specific primer was only positive for 45 isolates. This was further identified using the species-specific primer that target *Salmonella* Enteritidis 11 (24.4%), *Salmonella* Typhimurium 14 (31.1%) and other *Salmonella* spp. 20 (44.4%). In Oba Market, 1/4 (25%) were confirmed to be *Salmonella* Enteritidis, 1/4 (25%) were confirmed to be *Salmonella* Typhimurium, 2/4 (50%) were confirmed to be other *Salmonella* species (Table S5).

### Antimicrobial Susceptibility Profiles of the *Salmonella* Species From RTE Shrimps

The distribution of antimicrobial susceptibility profile of *Salmonella* species is presented in Table 1. For *Salmonella* Enteritidis, 100% (14/14) were resistant to erythromycin and penicillin, 85.7% (12/14) were resistant to amoxicillin/clavulanate and ampicillin, 92.9% (13/14) were resistant to amoxicillin, 71.4% (10/14) were resistant to ampicillin/sulbactam. For *Salmonella* Typhimurium, 100% (11/11) were resistant to erythromycin and penicillin, 90.9% (10/11) were resistant to ampicillin and amoxicillin, 72.7% (8/11) were resistant to amoxicillin/clavulanate, 63.6% (7/11) were resistant to doxycycline. For other *Salmonella* species, 100% (20/20) were resistant to erythromycin and penicillin, 80% (16/20) were resistant to amoxicillin, 75% (15/20) were resistant to ampicillin, 70% (14/20) were resistant to amoxicillin/clavulanate, 65% (13/20) were resistant to streptomycin. Number of resistant + intermediate *Salmonella* species as shown in Table 1 include 0/45 (cefotaxime, cephalothin, polymycin B and colistin), 45/45 (ampicillin, amoxicillin, erythromycin, penicillin, and amoxicillin/clavulanate), 38/45 (ampicillin/sulbactam), 37/45 (streptomycin), 36/45 (doxycline), 33/45 (tetracycline), 30/45 (oxytetracycline and ciprofloxacin), 26/45 (ofloxacin).

**Table 1:** Antimicrobial susceptibility profiles of the *Salmonella* species.

**Antimicrobial class**	**Antibiotics**	***Salmonella*** **species**								
		***Salmonella*** **Enteritidis (*****n*** **=** **14)**			***Salmonella*** **Typhimurium (*****n*** **=** **11)**			**Other** ***Salmonella*** **spp. (*****n*** **=** **20)**		
		**R**	**I**	**S**	**R**	**I**	**S**	**R**	**I**	**S**
Aminoglycosides	GEN	7.1	42.9	50	0	27.27	72.72	10	15	75
	KAN	0	21.4	78.6	0	9.09	90.9	0	5	95
	STR	50	28.6	21.4	54.5	27.27	18.18	65	20	15
	TOB	7.1	42.9	50	0	54.54	45.45	5	25	70
Aminopenicillins	AMP	85.7	14.3	0	90.9	9.09	0	75	25	0
	AMX	92.9	7.1	0	90.9	9.09	0	80	20	0
B-lactam/Beta-lactamase Inhibitors	SAM	71.4	14.3	14.3	45.45	54.54	0	50	25	25
Carbapenems	IPM	7.1	14.3	78.6	18.18	9.09	72.72	5	5	90
	MEM	0	7.1	92.9	0	9.09	90.9	0	10	90
Cephalosporins	CTX	0	0	100	0	0	100	0	0	100
	CEF	0	0	100	0	0	100	0	0	100
Folate pathway inhibitors	SUL	0	21.4	78.6	0	54.54	45.45	0	35	65
	TMP	0	7.1	92.9	0	27.27	72.72	0	10	90
Macrolides	ERY	100	0	0	100	0	0	100	0	0
Penicillins	PEN	100	0	0	100	0	0	100	0	0
	AMC	85.7	14.3	0	72.72	27.27	0	70	30	0
Phenicols	CHL	7.1	28.6	64.3	9.09	9.09	81.81	0	50	50
Ploymyxins	CST	0	0	100	0	0	100	0	0	100
	PMB	0	0	100	0	0	100	0	0	100
Tetracyclines	DOX	35.7	50	14.3	63.64	9.09	27.27	45	35	20
	OXY	42.9	21.4	35.7	27.27	45.45	27.27	35	30	35
	TET	50	28.6	21.4	36.36	18.18	45.45	50	30	20
Quinolone	CIP	50	35.7	14.3	36.36	18.18	45.45	25	35	40
	OFX	42.9	21.4	35.7	45.45	9.09	45.45	35	20	45

*GEN, Gentamycin (10 μg); KAN, Kanamycin (30 μg); STR, Streptomycin (25 μg); TOB, Tobramycin (10 μg); AMP, Ampicillin (10 μg); AMX, Amoxicillin (25 μg); SAM, Ampicillin/Sulbactam (30 μg); MEM, Meropenem (10μg); IPM, Imipenem (10 μg); CTX, Cefotaxime (30 μg); CEF, Cephalothin (30 μg); SUL, Sulfamethoxazole (30 μg); TMP, Trimethoprim (25 μg); ERY, Erythromycin (15 μg); PEN, Penicillin G (10 μunits); AMC, Amoxicillin/clavulanate (30 μg); CHL, Chloramphenicol (30 μg); CST, Colistin (20 μg); PMB, Polymyxin B (300 units); DOX, Doxycycline (30 μg); OXY, Oxytetracycline (30 μg); TET, Tetracycline (30 μg); CIP, Ciprofloxacin (10 μg); OFX, Ofloxacin (5 μg); R, Resistant; I, Intermediate; S, Sensitive*.

### Distribution of Multiple Antibiotic-Resistance Characteristics of the *Salmonella* Species

The MDR and MAR index distribution of *Salmonella* species is presented in Table 2. A total of 9/14 (64.3%) of *Salmonella* Enteritidis were resistant to 5 antibiotics (AMP^R^, AMX^R^, AMC^R^, ERY^R^, PEN^R^) which belonged to 3 different groups of antimicrobials with a MAR index of 0.21. Furthermore, 4/14 (28.6%) of *Salmonella* Enteritidis were resistant to 11 antibiotics (AMP^R^, AMX^R^, AMC^R^, STR^R^, SAM^R^, CIP^R^, OXY^R^, TET^R^, OFX^R^, ERY^R^, PEN^R^) which belonged to 8 different groups of antimicrobials with a MAR index of 0.46. A total of 9/11 (81.8%) of *Salmonella* Typhimurium were resistant to 4 antibiotics (AMP^R^, AMX^R^, ERY^R^, PEN^R^) which belonged to 3 different groups of antimicrobials with a MAR index of 0.17. Furthermore, 3/11 (27.3%) of *Salmonella* Typhimurium were resistant to 11 antibiotics (AMP^R^, AMX^R^, ERY^R^, PEN^R^, STR^R^, AMC^R^, DOX^R^, SAM^R^, TET^R^, CIP^R^, OFX^R^) which belonged to 7 different groups of antimicrobials with a MAR index of 0.46. A total of 9/20 (45%) of other *Salmonella* spp. were resistant to 6 antibiotics (STR^R^, AMP^R^, AMX^R^, ERY^R^, PEN^R^, AMC^R^) which belonged to 4 different groups of antimicrobials with a MAR index of 0.25. Furthermore, 3/20 (15%) of other *Salmonella* spp. were resistant to 12 antibiotics (STR^R^, AMP^R^, AMX^R^, ERY^R^, PEN^R^, AMC^R^, SAM^R^, DOX^R^, TET^R^, OXY^R^, OFX^R^, CIP^R^) which belonged to 7 different groups of antimicrobials with a MAR index of 0.50.

**Table 2:** Distribution of multiple antibiotic resistant characterizations of the *Salmonella* species.

***Salmonella* species**	**No of antimicrobial class**	**No of antibiotics**	**Resistance phenotypes**	**No of resistant species (%)**	**MAR index**
*Salmonella* Enteritidis (*n* = 14)	3	5	AMP^R^, AMX^R^, AMC^R^, ERY^R^, PEN^R^	9(64.3)	0.21
	6	8	AMP^R^, AMX^R^, AMC^R^, STR^R^, SAM^R^, CIP^R^, ERY^R^, PEN^R^	6(42.9)	0.33
	8	11	AMP^R^, AMX^R^, AMC^R^, STR^R^, SAM^R^, CIP^R^, OXY^R^, TET^R^, OFX^R^, ERY^R^, PEN^R^	4(28.6)	0.46
*Salmonella* Typhimurium (*n* = 11)	3	4	AMP^R^, AMX^R^, ERY^R^, PEN^R^	9(81.8)	0.17
	5	7	AMP^R^, AMX^R^, ERY^R^, PEN^R^, STR^R^, AMC^R^, DOX^R^	5(45.5)	0.29
	7	11	AMP^R^, AMX^R^, ERY^R^, PEN^R^, STR^R^, AMC^R^, DOX^R^, SAM^R^, TET^R^, CIP^R^, OFX^R^	3(27.3)	0.46
Other *Salmonella* spp. (*n* = 20)	4	6	STR^R^, AMP^R^, AMX^R^, ERY^R^, PEN^R^, AMC^R^	9(45)	0.25
	6	9	STR^R^, AMP^R^, AMX^R^, ERY^R^, PEN^R^, AMC^R^, SAM^R^, DOX^R^, TET^R^	8(40)	0.38
	7	11	STR^R^, AMP^R^, AMX^R^, ERY^R^, PEN^R^, AMC^R^, SAM^R^, DOX^R^, TET^R^, OXY^R^, OFX^R^	5(25)	0.46
	7	12	STR^R^, AMP^R^, AMX^R^, ERY^R^, PEN^R^, AMC^R^, SAM^R^, DOX^R^, TET^R^, OXY^R^, OFX^R^, CIP^R^	3(15)	0.50

*GEN, Gentamycin (10 μg); KAN, Kanamycin (30 μg); STR, Streptomycin (25 μg); TOB, Tobramycin (10 μg); AMP, Ampicillin (10 μg); AMX, Amoxicillin (25 μg); SAM, Ampicillin/Sulbactam (30 μg); IPM, Imipenem (10 μg); MEM, Meropenem (10 μg); CTX, Cefotaxime (30 μg); CEF, Cephalothin (30 μg); SUL, Sulfamethoxazole (30 μg); TMP, Trimethoprim (25 μg); ERY, Erythromycin (15 μg); PEN, Penicillin G (10 μunits); AMC, Amoxicillin/clavulanate (30 μg); CHL, Chloramphenicol (30 μg); CST, Colistin (20 μg); PMB, Polymyxin B (300 units); DOX, Doxycycline (30 μg); OXY, Oxytetracycline (30 μg); TET, Tetracycline (30 μg); CIP, Ciprofloxacin (10 μg); OFX, Ofloxacin (5 μg); R, Resistant; I, Intermediate; S, Sensitive; Values in parenthesis represent percentage; MAR, Multiple antibiotic resistance*.

### Distribution and Proportion of Virulence Gene Elements Among the *Salmonella* Species

The distribution of virulence genes among *Salmonella* species is presented in Table 3. For *Salmonella* Enteritidis 10/14 (71.4%) harbored *spiA* (involved in both biofilm formation and virulence), 11/14 (78.6%) revealed *sipB* (allows easy entering of non-phagocytic cells and lysing of macrophages), 14/14 (100%) harbored *inv*A (*Salmonella* invasion gene), 12/14 (85.7%) revealed *sifA* (for the development of filamentous assemblies) and *fljB* (flagellin gene), 13/14 (92.9%) harbored *sefA* (fimbrial subunit of *Salmonella* antigen) (Table 3).

**Table 3:** Distribution of virulence genes in the *Salmonella* species.

***Salmonella* species**	**Virulence determinants**					
	***spiA***	***sipB***	***invA***	***sifA***	***fljB***	***sefA***
*Salmonella* Enteritidis (*n* = 14)	10(71.4)	11(78.6)	14(100)	12(85.7)	12(85.7)	13(92.9)
*Salmonella* Typhimurium (*n* = 11)	9(81.8)	10(90.9)	11(100)	10(90.9)	10(90.9)	10(90.9)
Other *Salmonella* spp. (*n* = 20)	16(80)	15(75)	20(100)	18(90)	19(95)	18(90)
Total (*n* = 45)	35(77.8)	36(80)	45(100)	40(88.9)	41(91.1)	41(91.1)

### Distribution of Antibiotic-Resistant Elements Among the *Salmonella* Species

The distribution of antibiotic-resistant elements amongst *Salmonella* species is presented in Table 4. For *Salmonella* Enteritidis 9/14 (64.3%) harbored Class 1 integrase, 6/14 (42.9%) demonstrated Class 2 integrase, 8/14 (57.1%) revealed *sul*2 (sulphonamide resistance gene) and *catB*3 (group B chloramphenicol acetyltransferase gene), 10/14 (71.4%) revealed *flor* (florfenicol/chloramphenicol resistance gene), *tmp* (dihydrofolate reductase gene), *bla*_TEM_ (beta-lactamase resistant gene), 11/14 (78.6%) demonstrated *str*B (streptomycin inactivating enzyme), 12/14 (85.7%) harbored *dfr*1 (specific trimethoprim resistance), and *tet*C (tetracycline resistance protein) (Table 4).

**Table 4:** Distribution of antibiotic-resistant genes in the *Salmonella* species.

***Salmonella* species**	**Antibiotic-resistant genes**									
	**Class 1 integrase**	**Class 2 integrase**	***sul*2**	***flor***	***Tmp***	***str*B**	***dfr*1**	***bla*_**TEM**_**	***catB*3**	***tet*C**
*Salmonella* Enteritidis (*n* = 14)	9(64.3)	6(42.9)	8(57.1)	10(71.4)	10(71.4)	11(78.6)	12(85.7)	10(71.4)	8(57.1)	12(85.7)
*Salmonella* Typhimurium (*n* = 11)	7(63.6)	6(54.5)	7(63.6)	9(81.8)	10(90.9)	8(72.7)	10(90.9)	6(54.5)	4(36.4)	9(81.8)
Other *Salmonella* spp. (*n* = 20)	11(55)	5(25)	17(85)	16(80)	17(85)	16(80)	18(90)	12(60)	9(45)	17(85)
Total (*n* = 45)	27(60)	17(37.8)	32(71.1)	35(77.8)	37(82.2)	35(77.8)	40(88.9)	28(62.2)	21(46.7)	38(84.4)

## Discussion

Gastrointestinal illnesses continue to be a global and public health menace. Exposure to food borne *Salmonella* directly or indirectly via consumption of RTE seafood can be an important route of infection to humans. Findings from this study provide an estimation of the prevalence of *Salmonella* from RTE shrimps in open markets from south-south region in Nigeria. The prevalence of *Salmonella* positive samples was higher than a previous study from Turkey (Ikiz et al., 2016) (2%), Iran (Rahimi et al., 2013) (1.8%) and China (Yang et al., 2015) (13%). The prevalence of *Salmonella* spp. from the RTE shrimp samples assessed in this study was also lower compared to those detected from India (Kumar et al., 2008) (29.0%), Saudi Arabia (Elhadi, 2014) (39.9%), Vietnam (Nguyen et al., 2016) (49.1%), Thailand (Woodring et al., 2012) (21%), Brazil (Carvalho et al., 2013) (16.12%), China (Zhang et al., 2015) (29.7%) and India (Kumar et al., 2009) (26.7%); but higher than, findings by Koonse et al. (2005) from six different countries with participating countries not mentioned at their request (two countries are located in southeast Asia, one is in central Asia, one is in Central America, one is in North America, and one is an island in the Pacific Ocean) re-counted a prevalence rate of 1.6% in shrimp samples. It was also reported in Nigeria (Raufu et al., 2014) that a total of 23/200 (11.5%) samples were positive for *Salmonella*, with three serovars comprising *Salmonella* serovars Eko, 47: mt:-, and Hadar, recovered. In Brazil (Carvalho et al., 2013) reported that from a total of 186 confirmed *Salmonella* spp., five serovars were identified and they include: *Salmonella* Saintpaul, *Salmonella* Infantis, *Salmonella* Panama, *Salmonella* Madelia, and *Salmonella* Braenderup. Five different *Salmonella* serotypes including *Salmonella* Typhi, *Salmonella* Newport, *Salmonella* Paratyphi B, *Salmonella* Enteritidis, and *Salmonella* Typhimurium were recovered from seafood samples in Iran (Rahimi et al., 2013). The most prevailing *Salmonella* serovars from China (Zhang et al., 2015) among the 730 seafood samples examined were *Salmonella* Typhimurium (4.1%), *Salmonella* Hvittingfoss (4.1%), *Salmonella* Schwarzengrund (4.6%), *Salmonella* Stanley (4.6%), *Salmonella* Singapore (5.5%), *Salmonella* Thompson (9.2%), *Salmonella* Wandsworth (12.0%), and *Salmonella* Aberdeen (18.4%).

The findings from Yang et al. (2015) reported a most probable number (MPN)/g of 0.3–10, with one sample exceeding 110 MPN/g which was somewhat similar to the *Salmonella* density in this study. The mean *Salmonella* density in this study varied across the sampling months as higher densities were observed in the wet season (March to October) compared to dry season (November to February) and from one open market to another particularly from open markets in Delta State. Siala et al. (2017) reported that the presence of *Salmonella* spp. in shrimps is an indicator of contamination in the shrimp industry which happens to be one of the most significant seafood commodities worldwide. The high rate of positive *Salmonella* species in RTE shrimps in Southern Nigeria is worrisome and a substantial risk to public health. Thus, it is imperative to manage *Salmonella* infection in the food production process by further strengthening the surveillance of aquatic food products to circumvent the contamination of RTE seafood products. The high prevalence of *Salmonella* in open markets in the present study indicates poor sanitary condition during processing as well as the environment and poor hygiene of the RTE shrimp handlers during preparation of the products. The difference in the densities and prevalence of *Salmonella* from RTE seafood could also be ascribed to geographical variation, contaminated raw materials and poor /inadequate detection methods.

Determination of *Salmonella* resistance to antibiotics is crucial for therapeutic regimen during outbreaks. *Salmonella* resistance to erythromycin, amoxicillin and penicillin in this study are of public health threat and thus be as a consequence of extensive usage of these antibiotics in the study area. Interestingly, no *Salmonella* serovars was resistant to cefotaxime, cephalothin, colistin, and polymyxin B. This is very important to public health as these antibiotics could be crucial in threating drug resistant *Salmonella* pathogens. Public education to enlighten individual not to misuse these antibiotics is essential to circumvent the occurrence and development of resistance to these antibiotics.

Akiyama et al. (2011) reported from the United States that none of the *Salmonella* isolates showed resistance to ampicillin, gentamicin, chloramphenicol, kanamycin, sulfisoxazole, tetracycline, and streptomycin. The highest antibiotic resistance *Salmonella* species form seafood observed by Elhadi (2014) from Saudi Arabia were amoxicillin-clavulanic acid (45%), ampicillin (70%) and tetracycline (90.71%). Percentage resistance to nalidixic acid (47.4%) was the predominant report from Iran by Rahimi et al. (2013), prior to others such as ciprofloxacin (5.3%), trimethoprim (15.8%), streptomycin (15.8%), and tetracycline (36.8%). From China, Yang et al. (2015) reported resistance for ampicillin (28.2%), tetracycline (35.9%), trimethoprim-sulfamethoxazole (25.2%), streptomycin (18.4%) and chloramphenicol (20.4%), with 34.0% being resistant to more than three antibiotics. These were somewhat in accordance to the findings in this study. Zhang et al. (2015) also reported resistance of *Salmonella* from China from retail aquaculture products to tetracycline (34.1%), sulfonamides (56.5%), streptomycin (28.6%) and ampicillin (23.5%) with lower levels of resistance for ciprofloxacin (2.3%), gentamicin (3.2%), ceftazidime (0.5%) cefepime (0.5%), and cefotaxime (0.9%) which was rather similar to the findings in this study. In addition, 43.3% of the *Salmonella* serovars from a finding of Zhang et al. (2015) were multidrug resistant which is reduced when compared to the results in this study. *Salmonella* serotypes such as Typhimurium and Enteritidis have historically been reported as the significant causes of non-typhoidal salmonellosis. Though, other serotypes have been revealed to be included to be prevalent with respect to difference in geographical regions (Brands et al., 2005).

The occurrence of resistance to ciprofloxacin in *Salmonella* serovars is of public health importance as it translates possible misuse in animals and over-prescription in humans. *Salmonella* isolates in this study that were resistant to ciprofloxacin were also observed to be multidrug resistant strains to other antibiotics which were in accordance to the finding of Vo et al. (2006) from the Netherlands. MDR *Salmonella* isolates in this study are prevalent in open markets, which necessitates that more attention be ascribed toward the control and supervision of antibiotic usage, particularly in human health care and agriculture divisions in Nigeria. Bacterial virulence is predisposed by both the occurrence of antibiotic resistance and virulence determinants. The advancement of *Salmonella* strains that are is based particularly on elements of biochemical and genetic mechanisms so as to heighten their survival via preservation of their antibiotic resistance genes. As regards the virulence determinants that were analyzed, *Salmonella* Enteritidis, *Salmonella* Typhimurium, and other *Salmonella* isolates represent a broader range of pathogenicity.

High MAR index was observed in this study which indicates high use/misuse of antibiotics in the study areas. MAR index of *Salmonella* isolates ranged from 0.14 to 0.45 for different seafood in a study by Budiati et al. (2013) in Malaysia. From Brazil, Carvalho et al. (2013) reported that 23% of *Salmonella* serovars were resistant to ≤ 1 antibiotic, 20% were resistant to ≤ 2 antibiotics while 3 strains showed multi-resistance characteristics. These were lower compared to the findings of this study. The rapid development of bacterial resistance is ascribed to the selective pressure of antibiotics via evolutionary responses as a consequence of natural selection.

The dissemination of resistant elements in natural ecosystem can alter as well as change the physiology and population dynamics of resident microbial populaces (Igbinosa and Odjadjare, 2015). The emergence of antibiotic resistant determinants in pathogenic *Salmonella* species has made it more problematic due to the pervasiveness of horizontal gene transfer which is the procedure where bacteria obtain elements/determinants from the environment (Thomas and Nielsen, 2005). Most antibiotic resistance genes are found on intregons, plasmids or transposons, which can be transferred and mobilized to other bacteria of different or the same species. Integrons have been reported to be involved in the acquisition of antibiotic resistance elements. Class 1 integrons which contains numerous resistance elements could play vital roles in the maintenance and spread of antibiotic resistance in *Salmonella* species both in the absence and presence of selective pressure as reported in India (Deekshit et al., 2012). Meng et al. (2011) from China documented that class 1 integron showed empty regions from strains in serotypes Choleraesuis isolated from seafood. Findings by Meng et al. (2011) also suggest the possible dissemination of class 1 integrons from foodborne pathogens to human inhabited bacteria through horizontal gene transfer.

The occurrence and dissemination of resistant elements to pathogenic and commensal bacteria of human origin as well as gene transfer in human intestinal microbiome have been reported (Slayers et al., 2004). Antibiotic resistance genes such as *tet*A and *cat*A1 were present in 60 and 57.52%, of *Salmonella* isolates, respectively in a study by Deekshit et al. (2012) from India. Adesiji et al. (2014) reported that of the 20 tetracycline resistant isolates from India, 20(100%) *tet*A, 6(30%) *tet*B, 7(35%) *tet*C, and 10(50%) *tet*G encoded resistant elements, respectively. Of 18 cotrimoxazole-resistant strains, 4(22.2%), 14(77.7%), and 18(100%) had *sul*3, *sul*2, and *sul*1genes, respectively (Adesiji et al., 2014). Deekshit et al. (2013) reported the occurrence of three antibiotic resistance determinants *sul*1, *tet*G, and *flo*R from seafood some of which were also detected in this study.

Virulence determinants are involved in bacterial pathogenicity, and their occurrence in *Salmonella* can result in salmonellosis (). Findings from this study revealed that isolates of *Salmonella* Enteritidis and *Salmonella* Typhimurium demonstrated a diverse range of pathogenicity elements, which makes these serovars more virulent toward consumers of the RTE shrimp products especially immunocompromised individuals. Antibiotic resistance phenotypes and determinants have also been reported to be positively correlated with *Salmonella* virulence (Turki et al., 2014). Infections as a consequence of antibiotic-resistant *Salmonella* with virulence potential have been reported to take longer to recover from by been frequently fatal, when compared with ailments caused by antibiotic-susceptible strains of *Salmonella* with virulent capabilities.

The *spi*A gene of *Salmonella* is essential for its virulence and biofilm formation in host cells (Romling et al., 2003; Socher et al., 2005; Dong et al., 2011; Col et al., 2013; Beshiru et al., 2018). The *sip*B gene is required by *Salmonella* to form functional pores during *Salmonella* infection of erythrocytes for entry into the host cell through the host cell plasma membrane (Miki et al., 2004). The *sip*B gene is referred to as trans-locators as they translocate *Salmonella* effector proteins into host cells (consumers of RTE shrimps) which can cause typhoid fever and gastroenteritis (Galan and Wolf-Watz, 2006). The *sip*B gene in *Salmonella* serovars induces apoptotic macrophage either by activating or inducing autophagy and disruption of mitochondria, or by binding the proapoptotic enzyme caspase-1 which results in the discharge of interleukin-1 beta active form (Myeni et al., 2013).

A significant step in the cycle of facultative pathogenic intracellular *Salmonella* serovars on RTE shrimps and by extension the consumers is the incursion of the cells via the intestinal mucosa. Amplification of nucleotide sequences within the *inv*A gene of *Salmonella* has been evaluated as a means of detecting invasive *Salmonella* serovars (). The *inv*A gene of the *Salmonella* species allows the bacteria to invade the host and initiate infection, thereby increasing the degree of pathogenicity of the isolates. PCR analysis of 15 virulence genes by Yang et al. (2015) from retail seafood in China showed that all 103 *Salmonella* isolates had at least 4 virulence genes (*mgt*C, *ssa*Q, *sii*D, *bcf* C, and *sop*B), where the loci that remains were unevenly distributed. In addition, isolates of *Salmonella* Typhimurium, *Salmonella* Enteritidis, and *Salmonella* Weltevreden displayed a broader range of pathogenicity elements when compared with other *Salmonella* serovars by Yang et al. (2015) which was evident in this study.

A significant number of *Salmonella* serovars from RTE shrimps in this study harbored the *sif* A gene. The *sif* A gene plays a crucial role in *Salmonella* virulence. The degree of pathogenicity by *Salmonella* lies predominantly on the phenotypic manifestation of effector proteins released into the bacterial cell. *Salmonella* gains enterance into eukaryotic cells and exist in a vascular section with which some effector proteins (e.g., *sif* A) are located (Zhao et al., 2015).

Flagellin occurrence on RTE shrimps is a significant external antigen for numerous species which aids *Salmonella* virulence. Considerable heterogeneity of sequence exist within alleles which codes for different flagellar antigen from a previous study by McQuiston et al. (2004) while alleles which encodes similar antigenic flagella were homologous, signifying that flagellin determinants may be beneficial to targets for the genotypic resolve of flagellar antigenic type. Fimbriae are an important factor in *Salmonella* survival and persistence in the host (Kaur and Jain, 2012). The *sef* A gene encodes the *Salmonella* Enteritidis fimbrial protein (Mirmomeni et al., 2008). Studies have also revealed that the *sef* A gene plays a significant part in the adhesion of *Salmonella* Enteritidis to biotic surfaces (Lopes et al., 2006). Akiyama et al. (2011) reported that all *Salmonella* strains were positive for 14 virulence genes (*sif* A, *spi*A, *inv*A, *sop*E, *spa*N, *sip*B, *msg*A, *iro*N, *pag*C, *prg*H, *org*A, *lpf* C, *tol*C, and *sit*C) and negative for three genes (*cdt*B, *spv*B, and *pef* A). Some of these genes detected by Akiyama et al. (2011) were also detected in this study. Antibiotic resistance is a major public health menace globally, and particularly persistent in developing countries, including Nigeria, where the problem of infectious disease is on the increase with decreased healthcare budget. Though the emergence and dissemination of antibiotic-resistant *Salmonella* is a significant concern to food processors, cinnamaldehyde and carvacrol which are effective plant-derived antimicrobials have been reported to inactivate antibiotic-resistant *Salmonella enterica* in oysters, buffer and celery (Ravishankar et al., 2010). Bacteriophages propose effective and highly specific bio-control of antibiotic-resistant *Salmonella* pathogens from RTE foods (Guenther et al., 2012). Although phage particles keep their infectious capabilities, they are immobilized freely by the RTE food, which result in loss of their capacity to infect and diffuse target cells. Short-chain fatty acids have found application in animal diets to manage pathogens with *Salmonella* serovars inclusive (Van-Immerseel et al., 2002). Another alternative to eliminating pathogens is the precise suppression of functions vital to cause infection in the host (Clatworthy et al., 2007). Gene regulation mechanism via quorum sensing, where bacteria regulate the manifestation of numerous genes in reaction to the occurrence of small signal molecules is also very crucial (Defoirdt et al., 2011).

Other management strategies for antibiotic resistance includes the following: limiting the non-therapeutic usage of antibiotics for agriculture; improved information to strengthen resolutions on standard therapeutic regimen, education, other actions, coupled with continuous monitoring and validating effectiveness of management strategies; strengthening infection control boards in hospitals; nutrient management and runoff control; and improved diagnostic procedures, which requires developmental variations and infrastructure upgrades, enhancements in microbiological laboratory equipment and personnel (Global Antibiotic Resistance Partnership - India Working Group, 2011; Pruden et al., 2013). These recommendations could assist in the reduced of antibiotic resistance, directly advance public health, advantageous to the populace and decrease pressure on healthcare system. Finally, enhancing the coverage and types of juvenile vaccines administered by government agencies would enormously decrease the disease burden and circumvent the misuse of antibiotics (Global Antibiotic Resistance Partnership - India Working Group, 2011).

## Conclusion

Findings indicate that RTE shrimps act as reservoirs in harboring multiple *Salmonella* strains. The recovered *Salmonella* serovars which exhibits multiple virulence and antibiotic resistance genes coupled with high MAR index constitute a risk to consumers. Hence, it is crucial to monitor the usage of antibiotics and hygiene status in processing and post-processing handling to circumvent the acquisition and dissemination of virulent *Salmonella* serovars. Furthermore, maintenance, and implementation of control measures such as good manufacturing practices (GMP), and hazard analysis and critical control point (HACCP) coupled with education of the RTE shrimp processors is necessary, for reducing and/or spreading *Salmonella* contamination.

## Author Contributions

AB and II carried out the sampling, laboratory procedures, data interpretation, and writing of the manuscript. EI conceptualized, designed, and supervised the research, contributed in the laboratory methodologies and data interpretation, as well as the writing of the manuscript. All authors have read and approved the manuscript.

### Conflict of Interest Statement

The authors declare that the research was conducted in the absence of any commercial or financial relationships that could be construed as a potential conflict of interest.
