# Supplementary material for: Prevalence of Antimicrobial Resistance and Virulence Gene Elements of Salmonella Serovars From Ready-to-Eat (RTE) Shrimps
Source: Front Microbiol. 2019 Jul 11;10:1613. doi: 10.3389/fmicb.2019.01613 (PMC6637730; doi:10.3389/fmicb.2019.01613)
Supplement: Supplementary file 1 [file Table_1.doc]

**Supplementary Files**

**Prevalence of antimicrobial resistance and virulence gene elements of *Salmonella* serovars from ready-to-eat shrimps**

Abeni Beshiru^1^, Isoken H. Igbinosa^2^ and Etinosa O. Igbinosa^1,3^*

^1^ Applied Microbial Processes & Environmental Health Research Group,

Department of Microbiology, Faculty of Life Sciences, University of Benin, PMB 1154, Benin City, Nigeria.

^2^ Department of Environmental Management and Toxicology, Faculty of Life Sciences, University of Benin, PMB 1154, Benin City, Nigeria.

^3^Sustainable Development Office, University of Benin, Private Mail Bag 1154, Benin City 300001, Nigeria

*Corresponding author e-mail: eigbinosa@gmail.com

**Running title**: Resistance and virulence elements of *Salmonella* serovars

**Table S1**: Primers used in the identification of *Salmonella* species

| **Target species** | **Primer name** | **Sequence (5' to 3')** | **Target gene** | **Annealing condition** | **Amplicon size (bp)** | **Reference** |
| --- | --- | --- | --- | --- | --- | --- |
| *Salmonella* genus | S. 16S Rdnaf | TGTTGTGGTTAATAACCGCA | 16S rDNA gene | 56 °C for 60 s | 574 | Ziemer and Steadham (2003) |
|  | S. 16S Rdnar | CACAAATCCATCTCTGGA |  |  |  |  |
| *Salmonella* Enteritidis | ENT-F | AAATGTGTTTTATCTGATGCAAGAGG | *Ent* | 60°C for 90 s | 299 | Saeki *et al.* (2013) |
|  | ENT-R | GTTCGTTCTTCTGGTACTTACGATGAC |  |  |  |  |
| *Salmonella* Typhimurium | STM4492-F | ACAGCT TGGCCTACGCGAG | *Stm*4492 | 60°C for 90 s | 759 | Saeki *et al.* (2013) |
|  | STM4492-R | AGCAACCGTTCGGCCTGAC |  |  |  |  |

**Table S2**: Primers used for antibiotic resistance genes in *Salmonella* isolates

| **Target gene description** | **Primer** | **Sequence (5' to 3')** | **Target gene** | **Annealing condition** | **Amplicon size (bp)** | **Reference** |
| --- | --- | --- | --- | --- | --- | --- |
| Genetic makers for integron | class 1-F | GGGTCAAGGATCTGGATTTCG | intI1 gene | 55°C for 30 s | Variable | Mazel *et al*. (2000) |
|  | class 1-R | ACATGGGTGTAAATCATCGTC |  |  |  |
| Genetic makers for integron | class 2 –F | CACGGATATGCGACAAAAAGGT | intI2 gene | 55°C for 30 s | Variable | Mazel *et al*. (2000) |
|  | class 2-R | GTAGCAAACGAGTGACGAAATG |  |  |  |
| Sulfamethoxazole resistance | sul2-F | AGGGGGCAGATGTGATCGC | *sul2* | 60.5°C for 1 min | 625 | Falbo *et al*. (1999) |
|  | sul2-R | TGTGCGGATGAAGTCAGCTCC |  |
| Florfenicol/Chloramphenicol resistance gene | flor-F | TTATCTCCCTGTCGTTCCAGCG | *floR* | 60.5 °C for 1 min | 526 | Iwanaga *et al*. (2004) |
|  | flor-R | CCTATGAGCACACGGGGAGC |  |
| Dihydrofolate reductase gene | tmp-F | TGGGTAAGACACTCGTCATGGG | *dfr18* | 60.5 °C for 1 min | 389 | Falbo *et al*. (1999) |
|  | tmp-R | ACTGCCGTTTTCGATAATGTGG |  |
| Encode streptomycin inactivating enzyme | strB-F | GGCACCCATAAGCGTACGCC | *strB* | 60.5°C for 1 min | 470 | Dalsgaard *et al*. (1999) |
|  | strB-R | TGCCGAGCACGGCGACTACC |  |
| specific trimethoprim resistance | dfr1-F | CGAAGAATGGAGTTATCGGG | *dfrA1* | 60.5°C for 1 min | 372 | Iwanaga *et al*. (2004) |
|  | dfr1-R | TGCTGGGGATTTCAGGAAAG |  |
| Beta-lactamase resistant gene | bla_TEM_-F | ATAAAATTCTTGAAGAC | *bla*_TEM_ | 55°C for 1 min | 1073 | Kim *et al.* (2013) |
|  | bla_TEM_-R | TTACCAATGCTTAATCA |  |
| Group B chloramphenicol acetyltransferase gene | catB3-F | TCAAAGGCAAGCTGCTTTCTGAGC | *catB3* | 58°C for 1 min | 566 | Kim *et al.* (2013) |
|  | catB3-R | TATTAGACGAGCACAGCATGGGCA |  |
| Tetracycline resistance protein | tetC-F | AACAATGCGCTCATCGT | *tetC* | 65°C for 1 min | 1138 | Kim *et al.* (2013) |
|  | tetC-R | GGAGGCAGACAAGGTAT |  |

**Table S3:** Primers used to detect virulence gene in *Salmonella* isolates

| **Target genes descriptions** | **Primer** | **Sequence(5’-3’)** | **Annealing condition** | **Size (bp)** | **References** |
| --- | --- | --- | --- | --- | --- |
| *spi*A gene is involved in both biofilm formation and virulence | *SpiA*-F  *SpiA*-R | CCAGGGGTCGTTAGTGTATTGCGTGAGATG  CGCGTAACAAAGAACCCGTAGTGATGGATT | 55°C for 30 s | 550 | Ochman *et al*. (1996) |
| Entering non-phagocytic cells and lysing of macrophages | *SipB*-F  *SipB*-R | GGACGCCGCCCGGGAAAAACTCTC  ACACTCCCGTCGCCGCCTTCACAA | 55°C for 30 s | 875 | Skyberg *et al*. (2006) |
| *Salmonella* invasion gene | *InvA*-F  *InvA*-R | ACAGTGCTCGTTTACGACCTGAAT  AGACGACTGGTACTGATCGATAAT | 56°C for 30 s | 244 | Chiu and Ou (1996) |
| Necessitated for the development of filamentous assemblies which contains glycoproteins within lysosomal membrane in between the epithelial cells | *sifA*-F  *sifA*-R | ATGCCGATTACTATAGGCAATGG  TTATAAAAAACAACATAAACAGCCG | 55°C for 30 s | 1011 | Hur *et al*. (2011) |
| Flagellin gene: Flagellin is described as the subunit protein that assembles into filaments of bacterial flagella | *fljB*-F  *fljB*-R | ATGGCACAAGTCATTAATACAAAC  ACGCAGTAAAGAGAGGAC | 53 °C for 30 s | 1515 | Shah *et al*. (2011) |
| The foremost fimbrial subunit of *Salmonella* antigen is encoded by the *sef*A determinant | *sefA*-F  *sefA*-R | GCAGCGGTTACTATTGCAGC  TGTGACAGGGACATTTAGCG | 55°C for 30 s | 310 | Mirzaie *et al*. (2010) |

**Table S4.** *Salmonella* mean counts from ready-to-eat shrimps from open markets in Edo and Delta states, Nigeria

| **Months** | **Markets** | | | | | | | | | | | | ***p*-value** |
| --- | --- | --- | --- | --- | --- | --- | --- | --- | --- | --- | --- | --- | --- |
|  | **Oba Market** | **New Benin Market** | **Jattu Market** | **Igarra Market** | **Ekpoma Market** | **Uromi Market** | **Sapele Market** | **Ughele Market** | **Ogbegonogo Market** | **Ashafor Market** | **Igbudu Market** | **Main Market, Oleh** |  |
| Nov., 2016 | 1.215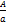 | 1.086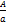 | 0.748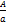 | 0.079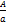 | 1.009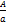 | 1.272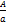 | 3.088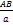 | 3.516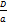 | 3.083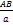 | 3.185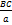 | 3.161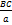 | 3.412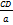 | 0.000 |
| Dec., 2016 | 0.977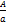 | 1.869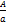 | 0.613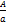 | 0.613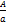 | 1.909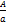 | 1.929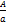 | 3.309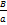 | 3.286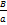 | 3.199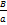 | 3.634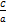 | 3.817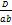 | 3.690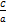 | 0.000 |
| Jan.,  2017 | 1.498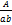 | 1.993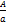 | 0.672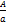 | 0.255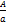 | 1.957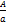 | 2.179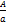 | 3.554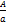 | 3.152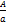 | 4.492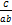 | 4.157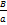 | 3.489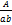 | 3.236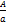 | 0.000 |
| Feb., 2017 | 1.982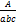 | 2.187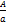 | 3.231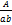 | 0.602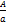 | 2.625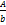 | 2.959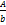 | 3.627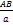 | 4.095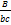 | 4.841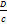 | 3.892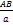 | 3.827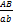 | 4.780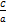 | 0.000 |
| Mar., 2017 | 2.391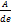 | 2.952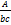 | 1.816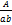 | 0.959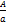 | 2.976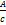 | 3.190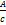 | 4.428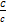 | 4.356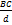 | 4.822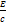 | 4.057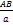 | 3.429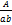 | 4.662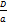 | 0.000 |
| Apr., 2017 | 2.458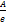 | 3.068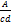 | 2.324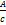 | 1.562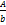 | 3.288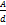 | 3.384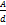 | 4.188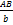 | 4.386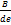 | 5.118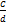 | 4.320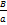 | 3.814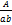 | 4.326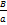 | 0.000 |
| May, 2017 | 2.669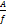 | 3.167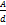 | 2.296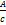 | 1.573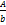 | 3.279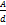 | 3.484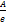 | 4.337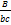 | 4.253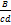 | 5.434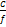 | 4.338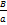 | 4.097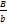 | 4.283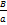 | 0.000 |
| Jun., 2017 | 2.185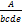 | 3.283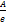 | 2.558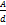 | 2.003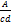 | 3.318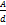 | 3.354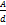 | 4.423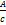 | 4.322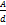 | 5.274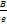 | 5.205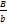 | 4.422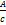 | 4.613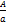 | 0.000 |
| Jul.,  2017 | 2.343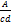 | 2.909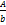 | 2.515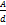 | 2.001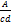 | 2.204 | 3.089 | 4.407 | 4.481 | 4.495 | 4.344 | 4.341 | 5.356 | 0.000 |
| Aug., 2017 | 1.782 | 2.209 | 2.023 | 1.966 | 2.045 | 2.495 | 4.330 | 4.337 | 4.278 | 3.836 | 4.435 | 4.555 | 0.000 |
| Sep., 2017 | 1.255 | 0.944 | 1.297 | 2.075 | 1.797 | 1.802 | 3.813 | 3.888 | 4.193 | 4.614 | 3.792 | 4.754 | 0.000 |
| Oct., 2017 | 1.093 | 1.814 | 0.813 | 1.297 | -0.301 | 1.552 | 2.572 | 3.053 | 3.748 | 3.431 | 3.283 | 3.371 | 0.000 |
| *p*-value | 0.000 | 0.000 | 0.000 | 0.000 | 0.000 | 0.000 | 0.000 | 0.000 | 0.000 | 0.000 | 0.000 | 0.000 |  |

**Legend**: Values are in log_10_ mean. Values with superscript which carry the same uppercase alphabets across columns show no significant difference while values in lowercase subscript which carry the same alphabets across rows also show no significant difference (*p*<0.05)

**Table S5**. Detection and distribution proportion of *Salmonella* species from ready-to-eat shrimps

| **Target species** | **Markets** | | | | | | | | | | | | **Total**  **(*n*=45)** |
| --- | --- | --- | --- | --- | --- | --- | --- | --- | --- | --- | --- | --- | --- |
|  | **Oba Market**  **(*n*=4)** | **New Benin Market**  **(*n*=4)** | **Jattu Market**  **(*n*=3)** | **Igarra Market**  **(*n*=3)** | **Ekpoma Market**  **(*n*=3)** | **Uromi Market**  **(*n*=4)** | **Sapele Market**  **(*n*=4)** | **Ughele Market**  **(*n*=4)** | **Ogbegonogo Market**  **(*n*=4)** | **Ashafor Market**  **(*n*=4)** | **Igbudu Market**  **(*n*=4)** | **Main Market, Oleh**  **(*n*=4)** |  |
| *Salmonella* genus | 4(100) | 4(100) | 10(100) | 10(100) | 10(100) | 4(100) | 4(100) | 4(100) | 4(100) | 4(100) | 4(100) | 4(100) | 45(100) |
| *Salmonella* Enteritidis | 1(25) | 1(25) | 1(33.3) | 1(33.3) | 1(33.3) | 1(25) | 2(50) | 0(0) | 1(25) | 1(25) | 1(25) | 0(0) | 11(24.4) |
| *Salmonella* Typhimurium | 1(25) | 1(25) | 1(33.3) | 1(33.3) | 1(33.3) | 0(0) | 1(25) | 2(50) | 1(25) | 1(25) | 2(50) | 2(50) | 14(31.1) |
| Other *Salmonella* spp. | 2(50) | 2(50) | 1(33.3) | 1(33.3) | 1(33.3) | 3(75) | 1(25) | 2(50) | 2(50) | 2(50) | 1(25) | 2(50) | 20(44.4) |

**References**

Ziemer, C.J., and Steadham, S.R. (2003). Evaluation of the specificity of *Salmonella* PCR primers using various intestinal bacterial species. Lett. Appl. Microbiol. 37, 463-469.

Saeki, E.K., Alves, J., Bonfante, R.C., Hirooka, E.Y., and Oliveira, T.C.R.M. (2013). Multiplex PCR (mPCR) for the detection of *Salmonella* spp. and the differentiation of the Typhimurium and Enteritidis serovars in chicken meat. J. Food Safety 33, 25-29.

Mazel, D., Dychinco, B., Webb, V.A., and Davies, J. (2000). Antibiotic resistance in the ECOR collection: Integrons and identification of a novel *aad* gene. Antimicrob. Agent. Chemother. 44, 1568-1574.

Falbo, V., Carattoli, A., Tosini, F., Pezzella, C., Dionisi, A.M., and Luzzi, I. (1999). Antibiotic resistance conferred by a conjugative plasmid and a class I integron in *Vibrio cholerae* O1 El Tor strains isolated in Albania and Italy. Antimicrob. Agents Chemoth. 43, 693-696.

Iwanaga, M., Toma, C., Miyazato, T., Insisiengmay, S., Nakasone, N., and Ehara, M. (2004). Antibiotic resistance conferred by a class I integron and *SXT* constin in *Vibrio cholerae* O1 strains isolated in Laos. Antimicrob. Agents Chemoth. 48, 2364-2369.

Dalsgaard, A., Forslund, A.N., Tam, D.X., Vinh, D.X., and Cam, P.D. (1999). Cholera in Vietnam: Changes in genotypes and emergence of class 1 integrons containing aminoglycoside resistance gene cassettes in *Vibrio cholerae* O1 strain isolated from 1979 to 1996. J. Clin. Microbiol. 37, 734-741.

Kim, M., Kwon, T.H., Jung, S.M., Cho, S.H., Jin, S.Y., and Park, N.H. (2013). Antibiotic resistance of bacteria isolated from the internal organs of edible snow crabs. PLoS ONE 8, e70887.

Ochman, H., Soncini, F.C., Solomon, F., and Groisman, E.A. (1996). Identification of a pathogenicity island required for *Salmonella* survival in host cells. Proc. Natl. Acad. Sci. U.S.A. 93, 7800-7804.

Skyberg, J.A., Logue, C.M., and Nolan, L.K. (2006). Virulence genotyping of *Salmonella* spp. with multiplex PCR. Avian Dis. 50, 77-81.

Chiu, C., and Ou J.T., (1996). Rapid identification of *Salmonella* Serovars in feces by specific detection of virulence genes, *inv*A and *spv*C, by an enrichment broth culture-multiplex PCR combination assay. J. Clin. Microbiol. 34, 2619-2622.

Hur, J., Choi, Y.Y., Park, J.H., Jeon, B.W., Lee, H.S., Kim, A.R., *et al.* (2011). Antimicrobial resistance, virulence-associated genes, and pulsed field gel electrophoresis profiles of *Salmonella enterica* subsp. enterica serovar Typhimurium isolated from piglets with diarrhea in Korea. Canadian J. Vet. Res. 75, 49-56.

Shah, D.H., Zhou, X., Addwebi, T., Davis, M.A., Orfe, L., Call, D.R., *et al*. (2011). Cell invasion of poultry-associated *Salmonella enterica* serovar Enteritidis isolates is associated with pathogenicity, motility and proteins secreted by the type III secretion system. Microbiol. 157, 1428-1445.

Mirzaie, S., Hassanzadeh, M., and Ashrafi, I. (2010). Identification and characterization of *Salmonella* isolates from captured house sparrows. Turk. J. Vet. Anim. Sci. 34, 181-186.
